# Aquafaba Hydrolysates as Functional Ingredients in Muffin Cakes: Effects on Physicochemical Properties, Quality Attributes, and Antioxidant Activity

**DOI:** 10.3390/foods14213709

**Published:** 2025-10-30

**Authors:** Hatice Bekiroglu

**Affiliations:** 1 Department of Food Engineering, Faculty of Agriculture, Sirnak University, Sirnak 73300, Türkiye; bkroglu@yildiz.edu.tr; Tel.: +90-212-373-4572; 2 Department of Food Engineering, Faculty of Chemical and Metallurgical Engineering, Yildiz Technical University, Istanbul 34220, Türkiye

**Keywords:** aquafaba, enzymatic hydrolysis, SDS-PAGE, texture properties, oxidative stability

## Abstract

Aquafaba, a legume cooking water typically discarded as waste, represents a sustainable and plant-based protein source with promising functional applications. In this study, aquafaba hydrolysates were produced by enzymatic treatment with flavourzyme and savinase, yielding two products with distinct degrees of hydrolysis (DH: ~10% and ~29%). Aquafaba hydrolysates obtained using flavourzyme (AFHs) and savinase (ASHs), together with aquafaba isolate (AI), were incorporated into muffin cakes as partial flour substitutes (5%). The addition of hydrolysates significantly influenced cake quality parameters, particularly antioxidant capacity and textural attributes. Enzymatic hydrolysis, particularly with savinase, produced the most pronounced functional improvements. Technologically, ASHs supplementation significantly enhanced cake expansion, with specific volume values (2.23 mL/g) nearly doubling compared to the control (1.04 mL/g). Crust color was markedly altered, with L* decreasing and a* and b* rising, reflecting darker, more browned surfaces due to intensified Maillard reactions. Both ABTS and DPPH assays demonstrated increased radical scavenging activity with higher DH, while SDS-PAGE confirmed the release of smaller peptide fractions. The ABTS radical scavenging activity of the control muffin (CM, 262.53 mg TE/100 g) significantly increased in AIM (muffin cake substituted with aquafaba protein isolate, 481.87 mg TE/100 g) and reached its highest values in muffins containing AFHs (489.74 mg TE/100 g) and ASHs (530.56 mg TE/100 g), respectively. Hardness, a critical quality parameter particularly relevant to storage stability, decreased in hydrolysate-enriched samples compared with both control and isolate formulations. Oxitest results showed that extended induction periods for hydrolysate-containing cakes (18:47 h) were longer relative to control muffins (15:08 h). Thermal analysis also indicated improved thermal stability in the presence of aquafaba. Overall, the findings demonstrate that aquafaba hydrolysates can be effectively utilized in bakery systems to enhance antioxidant activity, oxidative stability, and technological properties, while simultaneously contributing to sustainable food valorization.

## 1. Introduction

The global shift toward sustainable food systems has significantly influenced research and development within the food industry. The increasing demand for sustainable and plant-based food alternatives has propelled the exploration of novel ingredients that can replicate or enhance the functional properties of traditional animal-derived components [1,2]. Consumers increasingly seek functional, eco-conscious alternatives to animal-derived ingredients due to ethical, environmental, and health considerations [3]. In this context, aquafaba—a viscous liquid by-product from cooked legumes, primarily chickpeas—has gained recognition for its capacity to replicate the functional characteristics of egg whites, such as foaming, emulsification, and gelling [4]. Traditionally considered a by-product, aquafaba’s unique composition of carbohydrates, proteins, and other soluble plant solids has positioned it as a valuable resource in food formulation, aligning with the principles of waste reduction and resource optimization [5].

Recent advancements have explored the enzymatic hydrolysis of plant-based proteins to produce hydrolysates with enhanced functional and nutritional attributes [6,7,8,9]. Enzymatic hydrolysis is also gaining attention as an eco-friendly and sustainable approach for breaking down biomass or waste, as it functions under mild conditions, avoids toxic chemicals, offers higher specificity, lowers energy use, and contributes to circular economy efforts by turning waste into valuable products [10]. Enzymatic hydrolysis also involves the cleavage of protein molecules into smaller peptides and amino acids using specific enzymes, resulting in products with improved solubility, digestibility, and bioactivity [11,12]. These hydrolysates exhibit superior emulsifying and foaming capacities, making them suitable for incorporation into bakery products such as cakes and non-gluten baked goods to enhance texture, volume, and shelf-life [13,14,15,16].

In the context of bakery and cake products, the integration of aquafaba protein hydrolysates presents a promising avenue for innovation. Muffin cakes, characterized by their soft texture and rich flavor, can benefit from the functional properties of these hydrolysates. Studies have demonstrated that the inclusion of plant-based protein hydrolysates in cake formulations can lead to improved batter stability, increased volume, and enhanced crumb structure [17,18]. Moreover, the antioxidant properties inherent in certain hydrolysates contribute to the oxidative stability of baked goods, potentially extending their shelf-life [19,20,21]. While such studies are more common for protein isolates from legumes or cereals rather than aquafaba per se, they point to a method for enhancing the performance of plant protein-rich but dilute systems. Recent studies have extended the evaluation of aquafaba beyond egg substitution to its incorporation in baked goods. For example, Akbin et al. [22] compared cakes made with varying replacement levels of egg white using aquafaba from different legumes and assessed batter rheology, cake texture, volume, and related quality characteristics, using both conventional and microwave-infrared oven methods. They found that full replacement of egg white can reduce specific volume and change texture parameters, while partial replacement (e.g., ~50%) may still yield acceptable quality under certain baking conditions. Likewise, studies of eggless gluten-free cakes with aquafaba from different pulses have examined staling behavior, moisture retention, and radical scavenging activity during storage [23]. Given this background, there is a gap in knowledge concerning the integration of enzymatically hydrolyzed aquafaba proteins (i.e., aquafaba protein hydrolysates) into bakery products such as muffins. Muffin cakes were selected as a model bakery product due to their simple formulation, wide global consumption, and sensitivity to changes in ingredient functionality, making them suitable for evaluating the technological performance of protein hydrolysates. The application of aquafaba protein hydrolysates in muffin cakes not only addresses the demand for plant-based alternatives with lower allergenic potential but also contributes to the nutritional enhancement of these products.

This study aims to investigate the effects of incorporating enzymatically derived aquafaba protein hydrolysates into muffin cake formulations. In addition, by selecting flavourzyme and savinase—enzymes with different activity properties—for degradation, the study investigated in depth the effects of aquafaba hydrolysates with varying degrees of hydrolysis and peptide compositions on the basic quality characteristics of cake. By evaluating parameters such as physicochemical properties, batter rheology, baking performance, textural properties, and bioactive characteristics, the research seeks to elucidate the potential of these hydrolysates as functional ingredients in bakery applications. The findings are expected to contribute to the development of innovative, sustainable, and health-promoting baked goods, aligning with current trends in food science and consumer preferences.

## 2. Material and Methods

### 2.1. Material

The ingredients used in cake production, such as wheat flour (Sinangil Flour Company, Istanbul, Türkiye), sunflower oil, white granulated sugar (Torku Şeker, Konya, Türkiye), eggs, milk (UHT whole-fat cow milk (Icim, Ak Food Co., Sakarya, Türkiye)), vanilla, and baking powder (Kent Boringer, İstanbul, Türkiye) were purchased from a local market in Istanbul. Concentrated powdered aquafaba protein (78% purity, obtained from chickpea (*Cicer arietinum* L.)) was supplied by Döhler (Döhler, Istanbul, Türkiye). Savinase, flavourzyme, as well as reagents such as 2,4,6-tris(2-pyridyl)-s-triazine (TPTZ), 3-(2-pyridyl)-5,6-diphenyl-1,2,4-triazine-4′,4″-disulfonic acid sodium salt (ferrozine), 2,2-diphenyl-1-picrylhydrazyl (DPPH), and 2,2′-azinobis(3-ethylbenzothiazoline-6-sulfonic) acid (ABTS), were purchased from Sigma-Aldrich (St. Louis, MO, USA). All other chemicals and solvents used were of analytical grade.

### 2.2. Enzymatic Hydrolysis

Aquafaba protein was enzymatically hydrolyzed using two different enzymes, namely savinase and flavourzyme. Initially, two different 10% (*w*/*v*) aquafaba protein dispersions were prepared, and each enzyme was added to each at 1% (*w*/*w*) enzyme-to-substrate ratio. Hydrolysis with flavourzyme was performed at 50 °C and pH 7.0, while savinase treatment was conducted at 50 °C and pH 9.0. The pH of each reaction mixture was adjusted to the desired value using 0.1 N NaOH. The enzymatic reaction was maintained for 180 min under continuous agitation. To terminate hydrolysis, the mixtures were immediately heated to 100 °C for 15 min to deactivate the enzymes. Subsequently, samples were centrifuged at 15,000 rpm for 15 min at 4 °C to separate the hydrolysate. The resulting supernatants were then freeze-dried and stored in a desiccator at 4 °C until further analysis.

### 2.3. Degree of Hydrolysis (DH)

The extent of protein hydrolysis in aquafaba samples treated with savinase and flavourzyme was assessed using a modified pH-stat technique based on the method described by Adler-Nissen [24]. This approach maintains a stable pH throughout the enzymatic reaction by continuously neutralizing the hydrogen ions released during peptide bond cleavage. The process involves automated titration, where the volume of base (0.1 N NaOH) added to maintain the constant pH is directly proportional to the number of peptide bonds broken. The degree of hydrolysis (DH) was calculated using the following equations (Equations (1) and (2)).

(1) DH(%)=h/htot×100

(2) $$DH\left(\%\right)=B\times Nb\times 100/(\alpha \times Mp\times htot)$$

Here, *h* is the number of peptide bonds cleaved, calculated from the titration data; *htot* is the total peptide bonds per unit protein. The volume of base *B* and protein mass *Mp* were experimentally measured, while *Nb* and *α* are constants under the reaction conditions.

### 2.4. SDS-PAGE (Sodium Dodecyl Sulfate–Polyacrylamide Gel) Electrophoresis Analysis

The molecular weight distribution of aquafaba protein isolate and its hydrolysates was performed using SDS-PAGE according to the procedure described by Laemmli [25]. Protein samples were prepared at a concentration of 10 mg/mL in distilled water and were blended in equal volume (1:1, *v*/*v*) with a denaturing buffer composed of 0.5 M Tris–HCl (pH 6.8), 10% (*w*/*v*) sodium dodecyl sulfate (SDS), 0.5% (*w*/*v*) bromophenol blue, 5% (*v*/*v*) β-mercaptoethanol, and glycerol. The mixtures were then thermally denatured by incubation at 95 °C for 3 min. Aliquots (10 µL) of each denatured sample were loaded onto SDS-PAGE gels composed of a 4% (*w*/*v*) stacking gel and a 7.5–30% (*w*/*v*) gradient separating gel. Electrophoresis was performed in a Mini-PROTEAN^®^ vertical gel electrophoresis unit (Bio-Rad, Hercules, CA, USA) under a constant current of 20 mA per gel. Following separation, protein bands were visualized using 0.25% (*w*/*v*) Coomassie Brilliant Blue R-250 staining. Excess stain was removed using a destaining solution consisting of distilled water, methanol, and acetic acid in a 6:3:1 (*v*/*v*/*v*) ratio. Gel images were captured using a Gel Doc EZ imaging system (Bio-Rad, USA) for subsequent band analysis.

### 2.5. Muffin Preparation

The preparation of muffin samples was carried out as described in the previous study [20] with specific adjustments to incorporate aquafaba-derived ingredients. The complete formulation and proportions of ingredients are outlined in Table S1 (refer to Supplementary Materials). Initially, sugar, vanilla extract, shortening, and vegetable oil were blended in a high-speed stand mixer (KitchenAid, Benton Harbor, MI, USA) for 60 s. Subsequently, whole eggs were introduced into the mixture and blending continued for an additional 2 min to achieve a uniform consistency. In the next stage, wheat flour, baking powder, and milk were gradually incorporated into the batter. For enrichment purposes, 1% (*w*/*w*) of either aquafaba isolate (AI) or its enzymatically hydrolyzed forms (AFHs and ASHs) prepared using flavourzyme and savinase enzymes was used to partially replace wheat flour. The final batters were transferred into muffin molds and baked in a preheated electric oven (Maksan, Nevşehir, Türkiye) at 160 °C for 30 min, after preheating the oven for 15 min. The same oven was used for all batches to ensure consistent baking conditions. After baking, the muffins (see Figure S1, Supplementary Materials) were cooled at 20 ± 1 °C for approximately 3–4 h before undergoing physicochemical, antioxidant, and other quality evaluations. Each sample type was placed in the same position on the baking tray, and six muffin cakes were baked each time, with two repetitions.

### 2.6. The Flow Behavior of Muffin Batter

The flow behavior of muffin batters was examined using a controlled stress–strain rheometer (Anton Paar MCR 302, Graz, Austria) equipped with a 25 mm parallel plate geometry (PP25). Measurements were conducted at a constant temperature of 25 °C, maintaining a fixed gap of 1 mm between plates. Steady shear tests were performed across a shear rate range of 0.1 to 100 s^−1^. Experimental data were modeled using the Ostwald–de Waele (power law) equation to determine the consistency index (K, Pa·s^n^) and flow behavior index (n), which describe the viscous and flow characteristics of the batter.

(3) $$\pi =K\gamma^{n}$$

### 2.7. Some Physicochemical and Quality Properties of Muffins

The moisture content of muffin samples was determined using the air oven method according to the AACC method 44-15.02 [26,27]. Water activity (aw) in the muffin samples was assessed using a LabTouch-aw device (Novasina, Lachen, Switzerland). The total protein in muffin samples was quantified using the Kjeldahl method with a Behr Unit-S5 system (Ahlen, Germany). A nitrogen-to-protein conversion factor of 6.25 was employed, following the guidelines outlined by AACC [26]. To determine the oil content, the Soxhlet extraction method was applied using an E816 extraction system (Buchi, Flawil, Switzerland), ensuring accurate quantification of oil present in the samples [20]. The ash contents of the muffin samples were measured by incineration the sample in a muffle furnace set at 550 °C, according to the method outlined by AOAC [28]. All analyses were conducted under controlled conditions, with three replicates for each measurement to ensure accuracy and reproducibility across samples. The specific volume of muffin samples was assessed following the approach described by Ammar et al. [29] with modifications as necessary. This parameter was computed by dividing the total volume of each baked sample (measured in milliliters) by its corresponding mass (in grams), yielding a volume-to-weight ratio (mL/g) indicative of product aeration and structural quality. The color characteristics of the muffin samples were analyzed using a calibrated color measurement spectrophotometer (CR-400 Chroma Meter, Konica Minolta, Osaka, Japan) operated with a D65 standard light source and a 10° standard viewing angle. Measurements were carried out within the CIE L*, a*, b* color system, where colors are expressed as L* (lightness), a* (redness), and b* (yellowness). For each muffin, color measurements of both the crust (top surface) and crumb were taken at five distinct locations, with all measurements performed in at least three replicates.

### 2.8. Texture Profile Analysis

The textural properties of the muffin samples were evaluated using a texture analyzer (TA-XT2 Plus, Stable Micro Systems, Surrey, UK), fitted with a cylindrical compression probe (36 mm diameter), 5 kg of weight load, and a flat test platform. Analysis was performed by applying force to the upper crust region of whole cake samples and to the (crumb) inner part of each cake, which was prepared by carefully removing the top crumb part. Measurement conditions included the following: pre-test speed of 1.0 mm/s, test speed of 1.0 mm/s, and post-test (return) speed of 5.0 mm/s. The compression cycle generated a force–time curve, from which texture parameters were derived using the Texture Expert 1.05 software. The mechanical properties derived from the force–time curves included key texture attributes such as hardness, cohesiveness, adhesiveness, springiness, resilience, fracturability, gumminess, and chewiness [20]. All measurements were performed in at least three replicates to ensure accuracy and precision.

### 2.9. Oxidative Stability

The oxidative resistance of muffin samples was assessed according to the method described by Bekiroglu et al. [20] using an Oxitest oxidation testing chamber (Velp Scientifica, Usmate, MB, Italy). Approximately 10 g of each sample were loaded into the sealed reaction chamber of the instrument, which was then subjected to accelerated oxidative conditions. The test was conducted under a controlled temperature of 90 °C and an initial oxygen pressure of 6 bar. Throughout the analysis, oxygen consumption was monitored continuously until a marked decline in pressure indicated the onset of oxidation. The induction period (IP)—defined as the time point at which the oxidation rate significantly increases—was automatically recorded by the system and used as a measure of lipid oxidative stability.

### 2.10. Thermal Properties (Differential Scanning Calorimetry Analysis)

The thermophysical properties of the muffin formulations were characterized using a Differential Scanning Calorimeter (DSC; Q100, TA Instruments, New Castle, USA). The protocol was adapted with modifications from the method of Kemski et al. [30]. Approximately 10 ± 2.0 mg of finely homogenized muffin sample was sealed in an aluminum pan with hermetic lids to prevent moisture loss. Samples were subjected to a controlled heating program ranging from 25 °C to 300 °C at a constant ramp rate of 10 °C/min under an inert nitrogen atmosphere (50 mL/min) to eliminate oxidative interferences. An empty hermetically sealed aluminum pan served as the reference. Thermal transitions were analyzed from the resulting thermograms, and the onset temperature (T_0_), denaturation temperature (T_d_), and enthalpy change (ΔH) were computed from DSC curves using the instrument’s integrated analysis 2000 software (Version 4.3A, TA Instruments Ltd., New Castle, DE, USA).

### 2.11. Preparation of Muffin Extracts for Bioactivity Assessments

To prepare aqueous extracts from the muffin samples, 5 g of each sample were solubilized in 15 mL of distilled water and homogenized using a high-speed ultra-turrax homogenizer (Ultra-Turrax HG-15D, Daihan Scientific, Wonj, Republic of Korea) operating at 10,000 rpm for 5 min. These mixtures were incubated in a shaking water bath maintained at 30 °C for one hour to facilitate extraction. The homogenized mixtures were then subjected to centrifugation at 7500 rpm for 10 min using a centrifuge (Hettich Zentrifugen, Tuttlingen, Germany). The supernatant fraction was filtered through 0.45-micrometer syringe filters to remove any particulates and used for subsequent antioxidant capacity analysis [20].

### 2.12. Antioxidant Capacity Assays

#### 2.12.1. DPPH Assay

The antioxidant potential of muffin extracts was assessed based on the 2,2-diphenyl-1-picrylhydrazyl (DPPH) radical scavenging assay, with modifications from the protocol originally described by Brand-Williams [31]. A volume of 100 µL of each prepared extract was added to 4.9 mL of DPPH reagent solution (60 µM in methanol). The mixture was vortexed briefly to ensure homogeneity and then left to react for 30 min in the absence of light at ambient temperature. Following the incubation period, the absorbance of each sample was measured at 517 nm using a UV–visible spectrophotometer (Shimadzu UV-1800, Kyoto, Japan). Antioxidant activity was quantified by calculating the DPPH radical scavenging capacity and expressed as milligrams of Trolox equivalents per 100 g of sample (mg TE/100 g), using a standard curve generated with known concentrations of Trolox. All measurements were performed in triplicate with duplicate analyses to ensure reliability and reproducibility of the results.

#### 2.12.2. ABTS Assay

The antioxidant properties of muffin samples and aquafaba-derived hydrolysates were examined using the ABTS^+^ free radical scavenging method as adapted from Re et al. [32]. Initially, the ABTS stock solution was generated by dissolving ABTS to a final concentration of 7 mM in distilled water. To produce the ABTS^+^ radical cation, this solution was reacted with 2.45 mM potassium persulfate and left to incubate in darkness at room temperature for 16 h to ensure full radical development. Before analysis, the radical-containing solution was diluted with phosphate-buffered saline or distilled water until it reached an absorbance of 0.70 ± 0.02 at 734 nm. For the assay, 100 µL of sample extract was added to 2 mL of the prepared ABTS^+^ reagent. The mixtures were gently vortexed and allowed to stand for 6 min. The reduction in absorbance was then recorded at 734 nm using a UV–Vis spectrophotometer (Shimadzu UV-1800, Japan). The antioxidant efficacy was quantified by comparing the absorbance reduction to a Trolox standard curve, and results were reported as milligrams of Trolox equivalents per gram of sample (mg TE/g). Each measurement was conducted in triplicate with two analytical replicates.

### 2.13. Statistical Analysis

All data were statistically evaluated using SPSS software version 19.0 (IBM, Chicago, IL, USA). All values were expressed as means accompanied by their respective standard deviations. One-way analysis of variance (ANOVA) was used to assess differences between sample groups. Post hoc comparisons were conducted using Duncan’s multiple range test to identify statistically distinct means. Statistical significance was established at a confidence level of *p* < 0.05.

## 3. Result and Discussion

### 3.1. Degree of Hydrolysis (DH%)

**Figure 1:**
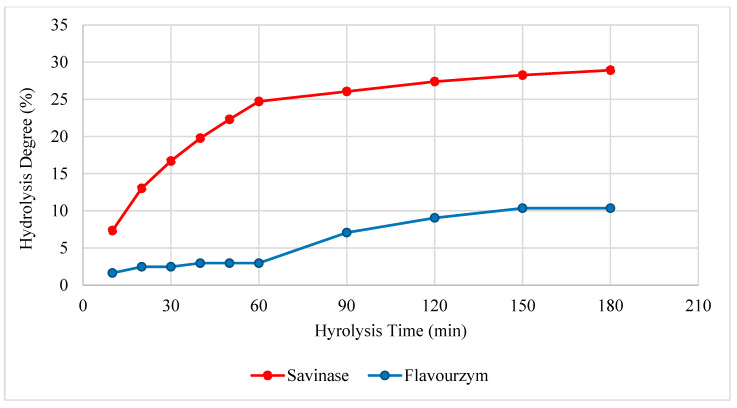
Degree of hydrolysis of aquafaba protein hydrolysates obtained by flavourzyme and savinase enzyme treatment.

The time-dependent hydrolysis degrees of aquafaba hydrolysates hydrolyzed under optimum pH and temperature conditions are shown in Figure 1. The enzymatic hydrolysis of aquafaba proteins for 180 min yielded markedly different degrees of hydrolysis (DH), with flavourzyme (AFHs) reaching ~10% and savinase (ASHs) achieving ~29%. The enzymatic activity of the enzymes for aquafaba protein varied considerably throughout the hydrolysis process. Savinase enzyme induced a rapid disintegration effect during the first 60 min, after which protein degradation proceeded at a relatively constant rate. Flavourzyme exhibited a lower degree of hydrolysis compared to savinase, characterized by an initial decline in the degradation rate during the first 60 min, followed by a progressive increase thereafter. This behavior suggests distinct enzymatic activity profiles and substrate interactions for the two enzymes. This variation is consistent with the inherent catalytic properties of the enzymes. Savinase, a subtilisin-type bacterial serine protease, is known for its broad and efficient endo-proteolytic activity, resulting in extensive peptide bond cleavage and higher DH values [33,34]. In contrast, flavourzyme derived from *Aspergillus* spp. combines both endo- and exo-peptidase activities and is generally expected to produce more free amino acids and short peptides. However, many studies have reported that flavourzyme produces much lower degradation and provides a limited hydrolysis effect compared to other enzymes [33,35,36,37]. This may be related to the generally narrower specificity of flavourzyme for endoprotease components [38].

### 3.2. Molecular Weight Profile (SDS-PAGE Analysis)

**Figure 2:**
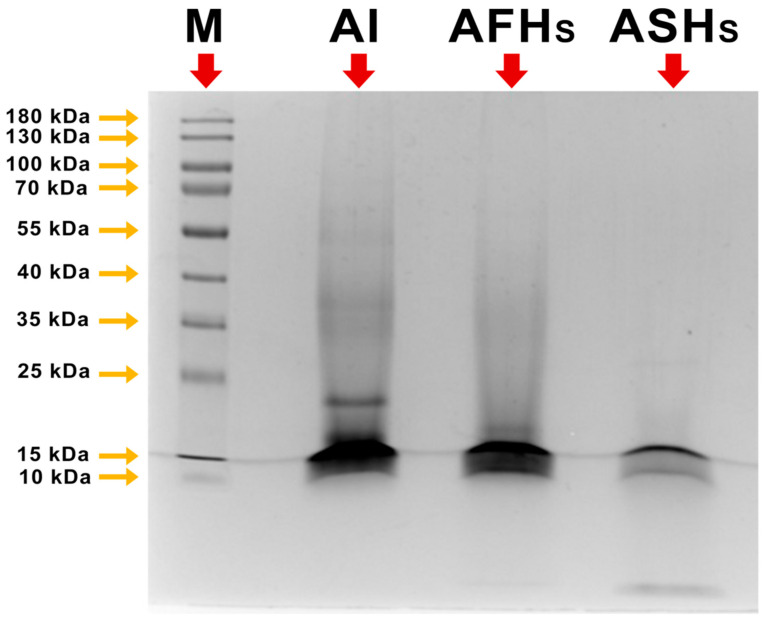
SDS-PAGE Image of aquafaba protein isolate and hydrolysates. M: Protein marker (Thermo Fisher Scientific, wide range 10,000–180,000 Da), AI: Aquafaba protein isolate, AFHs: Aquafaba hydrolysate obtained by flavourzyme enzyme treatment, ASHs: Aquafaba hydrolysate obtained by savinase enzyme treatment.

The protein molecular weight profile of aquafaba protein isolate (AI) and hydrolysates (AFHs and ASHs) is shown in the SDS-PAGE image (Figure 2). SDS-PAGE analysis revealed that the principal protein bands of sample AI, which was not subjected to enzymatic treatment, exhibited molecular weights ranging from approximately 6 to 100 kDa [39]. SDS-PAGE analysis indicated that enzymatic hydrolysis led to the disappearance of higher-molecular-weight bands and the concomitant appearance of new bands at lower molecular weights, reflecting protein cleavage and peptide formation. In AFHs, corresponding to a degree of hydrolysis of ~10%, the intensity of bands above 55 kDa markedly decreased, while faint smears were observed in the <10 kDa region, suggesting the formation of low-molecular-weight peptides. Although the molecular weight standards ranged from 10 to 180 kDa, peptides migrating below the lowest marker band were estimated to be <10 kDa based on their relative migration distance. Savinase treatment produced a markedly broader proteolytic effect, resulting in a degree of hydrolysis of ~29%. Consistent with this, SDS-PAGE analysis revealed a pronounced reduction in high-molecular-weight protein bands accompanied by the appearance of more abundant low-molecular-weight peptides, indicating extensive protein fragmentation. In the ASHs, almost all high-molecular-weight fractions were degraded, and the gel exhibited a diffuse smear in the low-molecular-weight region (<10 kDa), indicative of extensive proteolysis. These findings align with the stronger proteolytic activity of savinase compared with flavourzyme. Although studies investigating the molecular size distribution of peptides in aquafaba hydrolysates are limited, it is well-established that chickpea proteins are predominantly salt-soluble globulins, mainly composed of the vicilin (7S) and legumin (11S) storage proteins [40]. These globulins make up roughly 50–60% of the total seed protein [41]. While the protein profile of aquafaba may vary depending on its source, the predominant protein fraction is typically vicilin (7S), which is composed of three subunits—α′, α, and β—with approximate molecular weights of 80, 76, and 50 kDa, respectively [42]. Legumin (11S), the other major storage protein fraction, is a heterohexamer consisting of acidic and basic subunits with molecular weights of approximately 63.5 and 55 kDa, respectively [40]. In our SDS-PAGE of the intact isolate (AI), we observed bands in the region ~50–80 kDa and up to ~100 kDa, which likely correspond to vicilin and legumin subunits or their aggregates. Upon enzymatic hydrolysis, the disappearance of these higher-molecular-weight bands and the appearance of faint smears or bands below ~10 kDa is consistent with extensive proteolytic cleavage of these storage proteins into small peptides. Hydrolysis was found to degrade nearly all high–high-molecular-weight subfractions, especially those present in the intact protein (AI) structure.

### 3.3. Physicochemical and Color Properties of Muffin Cake Samples

Table 1 presents the physicochemical and color properties of muffin samples in which 5% of the flour was replaced with aquafaba protein isolate (AI) and with aquafaba hydrolysates (AFHs, ASHs) produced using flavourzyme and savinase enzymes. The control muffin (CM) sample showed about 21.9% moisture, 9.9% protein, and 12.83% oil, with water activity 0.77, and ash 1.56%. The muffin cake samples enriched with aquafaba substitutes (AIM, AFHM, ASHM) showed mean physicochemical properties as follows: moisture 21.2%, protein 12.4%, oil 12.6%, water activity 0.75, and ash 1.81%. Compared with the control sample, aquafaba substitution produced statistically significant changes (*p* < 0.05) in protein and ash contents. Specifically, protein content increased in the AFHM (12.29%) and ASHM (12.60%) treatments compared with CM (9.9%), confirming the nutritional enrichment effect. Ash content also increased significantly (1.81% vs. 1.56% in CM), reflecting mineral contributions from aquafaba. Moisture decreased slightly in AFHM (20.99%) and ASHM (21.06%) compared with CM (21.88%), but these differences were not statistically significant. Water activity and oil content also did not differ significantly among treatments. Although all cake samples were replaced with the same amount of isolate or hydrolysate, the fact that the protein contents were high in AHSM, which has a high degree of hydrolysis, can be associated with the increase in solubility in parallel with the increased hydrolysis [37]. As reported from many studies [11,38,43], enzymatic hydrolysis causes structural degradation, greater solubility, and recovery of more protein, thus increasing protein yield.

Specific volume, defined as the ratio of product volume to its mass (mL/g), is an important quality parameter for bread and other bakery products such as cakes, reflecting the extent of product expansion and internal structure development. Technologically, aquafaba supplementation significantly improved cake expansion, nearly doubling the specific volume (1.9–2.2 mL/g compared to 1.04 mL/g), indicating enhanced aeration and gas retention within the batter (Figure S1). In previous studies involving gluten-free flours or the use of aquafaba as an egg substitute, aquafaba addition was generally associated with a reduction in specific volume [44,45,46,47]. In contrast, our study, in which aquafaba was incorporated into wheat flour for enrichment, demonstrated an increase in specific volume. The highest specific volume was observed at a significantly higher hydrolysis degree (DH) compared to AHSM. Aquafaba, obtained from chickpeas, exhibits superior foaming and emulsifying properties compared to other legume-derived extracts, largely due to its unique composition of soluble proteins (mainly globulins and albumins) and polysaccharides such as galactomannans and arabinogalactans [48]. Enzymatic hydrolysis further enhances these functional properties by improving the foaming ability of aquafaba proteins, thereby increasing their gas-holding capacity and contributing to greater specific volume in bakery products such as cakes [49,50]. Crust color parameters also showed significant differences: Color parameters changed when different aquafaba products were added. L* and b* were generally lower, while a* values were higher compared with the control, indicating darker and more intensely browned crusts, consistent with enhanced Maillard reactions due to added peptides and amino acids (Figure S1). The crust L* values of the cakes decreased with the addition of AI, AFHs, and ASHs and this change was observed as 69.12 (CM), 63.96 (AIM), 58.80 (AFHM), and 57.06 (ASHM), respectively (*p* < 0.05). In contrast, crumb color was only slightly but still significantly affected, showing minor reductions in L* and modest shifts in a* and b*. These results suggest that aquafaba isolate and enzyme hydrolysates, despite causing stronger surface browning, provide statistically significant improvements in protein enrichment and volume, making them promising functional ingredients in cake formulations [51,52]. The significant changes in color values observed with the addition of AI, compared to the control cake, followed a similar trend to those obtained with AFHs and ASHs, indicating that color differences were associated with the increasing degree of hydrolysis. Consistent with our findings, the use of aquafaba in sponge cake production has been reported to result in a significantly darker crust color, primarily due to intensified caramelization and Maillard reactions [53]. This effect is attributed to the higher levels of simple carbohydrates and monomeric compounds in aquafaba compared to egg whites.

### 3.4. Rheological Properties of Muffin Batter

**Figure 3:**
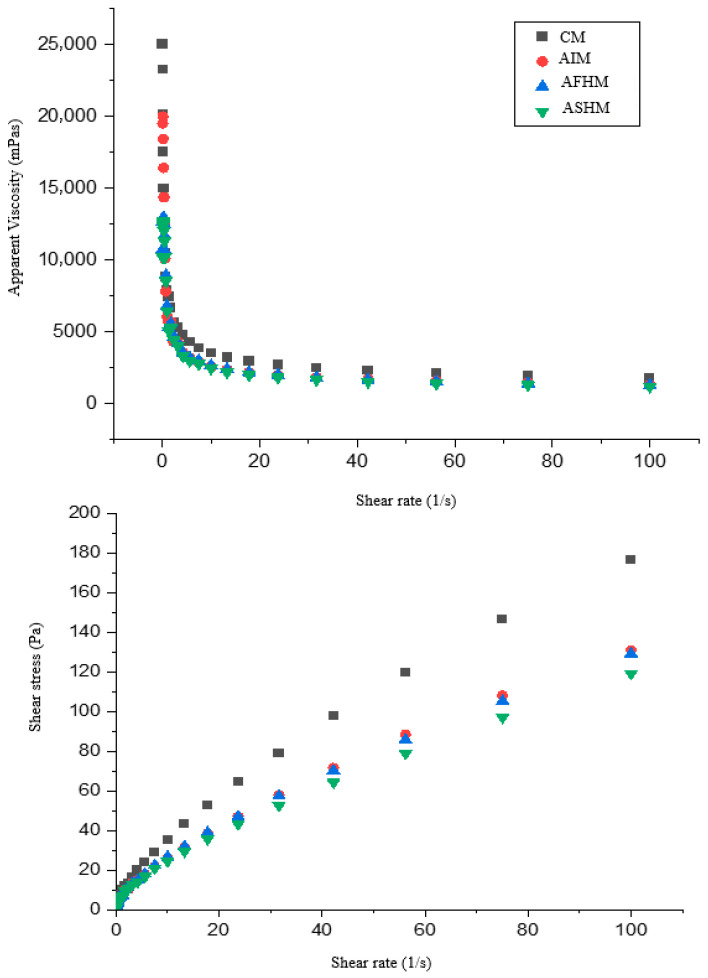
Rheological profiles of muffin cake batters. CM: Control muffin cake, AIM: Muffin cake substituted with aquafaba protein isolate, AFHM: Muffin cake substituted with aquafaba hydrolysate obtained by flavourzyme enzyme treatment, ASHM: Muffin cake substituted with aquafaba hydrolysate obtained by alcalase enzyme treatment.

The rheological properties of cake batter samples substituted with aquafaba protein isolate (AI) and hydrolysates (AFHs and AFHs) were characterized as shown in Table 2 and Figure 3. The control sample (CM) has the highest K value (9.03). This indicates that it has the highest viscosity and that the mixture has a denser structure. A significant decrease in the K value is observed with the addition of aquafaba isolate (AIM) and especially enzyme hydrolysates (AFHM, ASHM). In other words, the breakdown of protein with enzyme hydrolysate has caused the dough to become more fluid. Since n < 1 in all samples, the cake batters are pseudoplastic fluids exhibiting shear thinning [54]. The AIM sample has the lowest n value at 0.579; thus, this sample exhibits the most pronounced shear thinning behavior in response to shear rate. In enzyme hydrolysate samples (AFHM: 0.625, ASHM: 0.615), the n values are slightly higher, meaning they exhibit a more stable fluidity behavior. For the flavourzyme hydrolysate (DH: ~10%), due to its lower degree of hydrolysis, the partial breakdown of the protein has facilitated the flow of the dough (K has decreased), but by increasing the n value, it has ensured a more consistent flow behavior. With a higher degree of hydrolysis for ASHs (DH: ~29%), it has broken down the proteins more, causing the viscosity to decrease further (K = 6.39). However, according to AIM, the n value remained slightly higher, meaning it exhibited a more balanced rheological profile. The isolate form of aquafaba protein increases the viscosity of the dough, while enzyme hydrolysates reduce this viscosity and make the dough more fluid. ASHs, in particular, produced the lowest K value due to its high degree of hydrolysis. However, since all samples exhibited shear thinning behavior, a suitable structure was maintained in terms of processability in the cake formulation (Table 2).

In all samples, viscosity decreased as the shear rate increased, meaning that the doughs exhibited shear thinning (pseudoplastic) behavior (Figure 3). The control (CM) had the highest viscosity, while the enzyme hydrolysates (AFHM, ASHM), in particular, exhibited lower viscosity. This indicates that addition of hydrolyzed protein made the dough more fluid. Shear stress increases with shear rate; while the control sample yielded the highest values, enzyme-hydrolyzed doughs exhibited lower shear stress. Although the differences between AIM, AFHM, and ASHM are small, the lowest values are generally found in ASHM. The control dough is denser and more viscous, while the aquafaba isolate and especially the enzyme hydrolysates made the dough more fluid (Figure 3). Previous studies have demonstrated that the incorporation of proteins and their hydrolysates into cake batters can markedly influence batter flow properties [55]. Consistent with our findings, Sung et al. [56] reported that the addition of soy protein concentrate (3–24%) to sponge cake led to a decrease in viscosity proportional to the increase in protein content. This may also be associated with the increased oil and water-binding capacity of protein hydrolysates compared to intact proteins.

### 3.5. Texture Profile Analysis

Texture is a key organoleptic attribute of foods, particularly bakery products such as cakes, and represents one of the most critical factors influencing consumer preference and overall product acceptance [57]. Therefore, it is essential that additives intended for bioactive or functional enrichment also support and enhance the structural properties specific to the product. The texture properties of cake samples were characterized to evaluate the effects of aquafaba protein isolate and enzymatic hydrolysates on product quality. Muffin cake samples were evaluated based on textural parameters, including hardness, springiness, cohesiveness, gumminess, chewiness, and resilience, each reflecting distinct aspects of texture quality.

The hardness value—defined as the force required to deform the product by a given distance—decreased in cake samples containing AI substitution and was further reduced with the addition of AFHs and ASHs (Table 3). Overall, cake hardness tended to decrease as the degree of hydrolysis increased and with the incorporation of aquafaba protein. This inverse relationship was observed consistently across all samples, and the changes were significant compared with the control (*p* < 0.05). The hardness value, measured at 4043 g in the control cake (CM) prepared with only wheat flour, decreased to 3511 g in the AIM and further to 2828 g and 2317 g in the cakes enriched with flavourzyme and savinase hydrolysates, respectively. In a related study, Ozón et al. [58] enriched bread with bioactive peptides derived from chia (*Salvia hispanica* L.) expeller and reported that hardness values decreased (2162 g) significantly with increasing concentrations of chia expeller hydrolysates compared to the control bread (2986.4 g) prepared solely with wheat flour. In another study, it was determined that adding hydrolysate to bread formulations caused a decrease in firmness. Karimi et al. [59] attributed this to the competition between hydrophilic groups in the hydrolysates and starch molecules for water molecules during gelatinization, and the resulting incomplete gel structure leading to a softer dough.

The ability of hydrolysates to enhance gas retention in cake batter, increase specific volume, and promote a softer crust structure can be attributed to improved technological functionalities, such as emulsifying capacity and oil- and water-binding properties, which arise from protein degradation. Similar trends were observed in gumminess values—which reflect the energy required to prepare a semi-solid food for swallowing—as in the hardness values of the cake samples [60]. The gumminess value of the CM was 2323 g, which showed a slight, non-significant reduction in the AIM (2313 g). In contrast, the adhesiveness of the AFHM (1985 g) and ASHM (1516 g) decreased significantly, correlating with the increasing degree of hydrolysis (*p* < 0.05). Oprea et al. [61] investigated the impact of fish protein hydrolysate on bread quality during storage and reported that gumminess decreased as the proportion of hydrolysate in the formulation increased (from 1.07 N to 0.67 N). Although the addition of aquafaba resulted in significant changes in parameters such as springiness, cohesiveness, gumminess, and resilience compared to the control sample, the incorporation of hydrolysates did not produce a statistically significant effect on these properties. These results indicate that enzymatic hydrolysis, particularly, enhanced textural quality, likely through the formation of shorter peptides with superior solubility, water-binding capacity, and emulsifying activity that stabilized batter aeration and crumb structure during baking [20,62]. Taken together, these findings also suggest that controlled enzymatic hydrolysis of aquafaba proteins, and particularly the choice of enzyme, can be strategically applied to enhance the texture of baked products without compromising structure or quality. Moreover, the literature reports that enzymatically hydrolyzed proteins exhibit improved emulsifying and foaming capacities, which in turn support increased specific volume and finer texture in bakery applications [63].

### 3.6. Thermal Properties (Differential Scanning Calorimetry Analysis)

**Figure 4:**
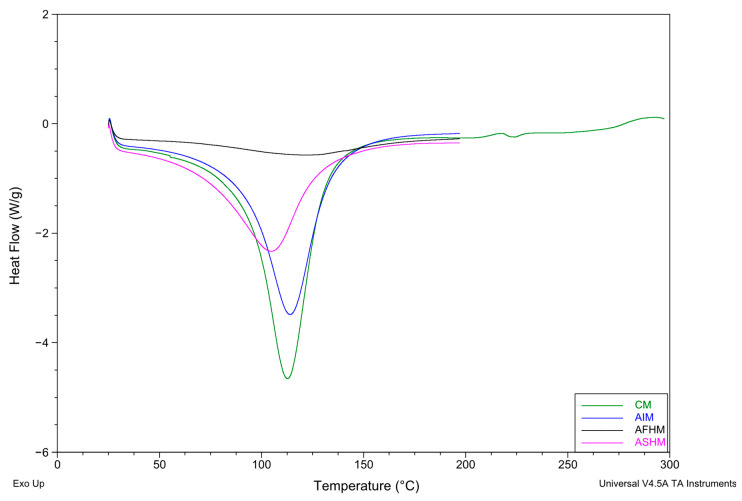
Thermal properties of muffin cake samples. CM: Control muffin cake, AIM: Muffin cake substituted with aquafaba protein isolate, AFHM: Muffin cake substituted with aquafaba hydrolysate obtained by flavourzyme enzyme treatment, ASHM: Muffin cake substituted with aquafaba hydrolysate obtained by alcalase enzyme treatment.

DSC analysis showed that aquafaba isolate and its hydrolysates significantly modified the thermal properties of muffin cakes (Figure 4) (*p* < 0.05). Control muffins (CMs) showed an onset temperature of 95.18 °C, a denaturation temperature of 114.46 °C, and an enthalpy of 329.7 J/g. With aquafaba isolate (AIM), T_0_ (94.66 °C) and T_d_ (114.53 °C) were similar, but ΔH increased to 360.9 J/g, suggesting a more stable protein matrix. Muffins containing flavourzyme hydrolysate (AFHM; DH: ~10%) had a lower T_0_ (70.53 °C), a higher T_d_ (122.34 °C), and reduced ΔH (270.63 J/g). This indicates that partial hydrolysis created flexible structures initially, which reorganized during heating into more resistant aggregates. Savinase hydrolysate muffins (ASHM; DH: ~29%) showed both lower T_0_ (75.10 °C) and T_d_ (105.49 °C), together with reduced ΔH (283.3 J/g), reflecting extensive proteolysis and loss of structural integrity. These results suggest that extensive proteolysis disrupted structural integrity more profoundly, leading to smaller peptides and diminished stability. Taken together, aquafaba isolate consistently enhanced thermal behavior, while hydrolysates exerted degree-dependent effects: moderate hydrolysis (~10%) promoted partial stabilization through peptide interactions, whereas deeper hydrolysis (~29%) weakened the overall thermal network. In a study on the thermal stability of soy protein and its hydrolysates, findings consistent with our results were reported: compared with the isolate, soy hydrolysates with a higher degree of hydrolysis exhibited markedly lower ΔH. This reduction was attributed to extensive degradation of the structural subfractions (7S globulin and 11S acidic polypeptide subunits) present in the hydrolysates [64]. Dent et al. [13] reported that the thermal stability of chickpea and soy proteins obtained with alcalase and flavourzyme treatments was lower than that of intact protein samples, and that this thermal stability loss varied depending on the enzyme–substrate interaction.

### 3.7. Oxidative Stability

The results of the oxidation test for muffin cake samples, obtained under accelerated analytical conditions (at 90 °C and 6 bar), are presented in Table 4. The induction period (IP) values, used as indicators of the oxidative stability of the cakes, were determined to be 15:08 h, 16:32 h, 17:28 h, and 18:47 h for the CM, IM, AFHM, and ASHM samples, respectively. Among the formulations, incorporation of savinase-derived aquafaba hydrolysate (ASHs; DH: ~29%) conferred the most substantial oxidative protection, reflecting both the highest radical scavenging potential and the most pronounced suppression of lipid peroxidation. This superior performance highlights the critical role of extensive proteolysis in liberating bioactive peptide fractions with electron-donating and chain-breaking capacities [34]. In contrast, cakes containing flavourzyme-derived hydrolysate (AFHs; DH: ~10%) demonstrated moderate antioxidant functionality, while those enriched with aquafaba isolate or prepared solely with wheat flour showed progressively weaker resistance to oxidative deterioration. Protein hydrolysates generated through enzymatic hydrolysis are widely recognized as a promising source of antioxidant peptides [65,66]. This ranking is consistent with the well-established relationship between enzyme specificity, degree of hydrolysis (DH), and peptide release. Broad-spectrum serine endopeptidases such as savinase promote deeper proteolysis, yielding low-molecular-weight, hydrophobic, and electron-donating peptides with strong radical scavenging activity [67]. This explains the superior antioxidant performance of ASHM cakes in both DPPH/ABTS assays and in reducing lipid oxidation products. Recent studies reported that hydrolysates, exhibiting markedly higher hydroxyl radical scavenging activity than intact proteins, can be employed to inhibit lipid peroxidation in foods [68,69]. The incorporation of protein hydrolysates into food products can enhance technological and functional attributes while also improving antioxidant capacity and extending shelf-life. Previous studies have demonstrated the pronounced oxidative stabilization effect of protein hydrolysates in fortified food products [70,71]. These findings can be attributed to the release of amino acid residues during enzymatic hydrolysis which become active and available to effectively inhibit the oxidation of unsaturated fatty acids [72]. In addition, polypeptides released during hydrolysis can surround lipid molecules and protect lipid droplets from oxidation by forming a physical barrier [72]. Protein hydrolysates generated through enzymatic hydrolysis are widely recognized as a promising source of antioxidant peptides [65,66].

### 3.8. Antioxidant Capacity

The results of the antioxidant capacity analyses obtained by the DPPH and ABTS methods are presented in Table 4. The antioxidant capacity of various natural substances is frequently evaluated using the DPPH method, in which the radical accepts a hydrogen donor from antioxidants, leading to the cessation of oxidative chain reactions [65,73]. The DPPH radical scavenging capacity of aquafaba protein samples (AI, AFHs, and ASHs) ranged from 11.7 to 26.41 mg TE/g sample. Among them, the untreated isolate (AI) exhibited the lowest activity, while enzymatic hydrolysis significantly enhanced the antioxidant capacity, yielding 18.97 mg TE/g in AFHs and 26.41 mg TE/g in ASHs (*p* < 0.05). The ABTS radical scavenging assay is widely applied to evaluate the antioxidant capacity of both hydrophilic and lipophilic compounds, owing to its solubility in aqueous and organic media and its applicability across a broad pH range [74,75]. AI and its hydrolysates were more effective at reducing ABTS radicals than DPPH radicals. This can also be explained by the higher reactivity and lower stability of the ABTS radical compared to the DPPH radical [76]. This scavenging power against the ABTS radical was determined as 222.58, 257.01, and 294.24 mg TE/ g sample for AI, AFHs, and ASHs, respectively. Enzymatic hydrolysis treatment produced a remarkable effect (*p* < 0.05) on the antioxidant capacity of aquafaba, and this effect was further strengthened with an increasing degree of hydrolysis. This increase can be attributed to the release of low-molecular-weight peptides and phenolic–peptide complexes during enzymatic hydrolysis, which are known to be highly effective in neutralizing free radicals [77]. Numerous studies have demonstrated that the effects of enzymatic hydrolysis are strongly associated with the degree of hydrolysis, highlighting its critical role in determining functional and nutritional properties [66,78]. For instance, in chickpea protein hydrolysis with alcalase and flavourzyme, the greatest DPPH radical scavenging activity (48.4%) was observed in the hydrolysate produced by their combined application, which also exhibited the highest degree of hydrolysis (50.2%) [37]. Likewise, Jamdar et al. [79] found that elevating the hydrolysis degree of peanut protein from 10% to 20% increased DPPH radical scavenging activity from 21% to 51% at 2.0 mg/mL. The impact of enzymatic hydrolysis and its degree were likewise evident in the cake samples. Enzymatic hydrolysis of aquafaba markedly enhanced the radical scavenging capacity of the enriched muffins (Table 4). Both DPPH and ABTS assays showed significant increases versus the control, consistent with greater concentrations of low-molecular-weight peptides and liberated phenolic–peptide complexes that form during proteolysis and are effective radical quenchers [80]. The DPPH radical scavenging capacity was lowest in the CM (51.01 mg TE/100 g sample) and increased significantly with the addition of the aquafaba protein isolate (AIM-84.35 mg TE/100 g sample). This enhancement was further observed in AFHM (105.46 mg TE/100 g sample) and ASHM (115.46 mg TE/100 g sample) cakes in that order (*p* < 0.05). The ABTS radical scavenging activity of all cake samples exhibited a similar trend, showing markedly higher values compared to DPPH. The antioxidant activity increased significantly with AI substitution compared to the control cake (262.53 mg TE/100 g sample), showed a further increase in AFHM (489.74 mg TE/100 g sample), and reached the highest level in the ASHM (530.56 mg TE/100 g sample) sample, which exhibited the greatest degree of hydrolysis. Savinase treatment facilitated extensive hydrolysis of aquafaba proteins, producing smaller peptides with relatively lower molecular weights [33,67,81]. These peptides, generated by enzymes such as savinase or flavourzyme, expose free thiol groups and amino acid residues (e.g., Tyr, Trp, His, Met) capable of donating hydrogen atoms or electrons to neutralize DPPH and ABTS radicals [82]. This mechanism accounts for the stronger antioxidant activity observed in the hydrolysates compared with the control and α samples in both assays, even after thermal processing such as baking. Although aquafaba hydrolysates have not yet been extensively investigated, numerous studies have reported that protein hydrolysates from various sources contribute to enhanced antioxidant properties in bakery products [20,58,61]. Mohammadi et al. [21] demonstrated that the enrichment of muffins with lentil proteins and their hydrolysates markedly enhanced antioxidant activity (~60–66% radical scavenging). The improvement was more pronounced with the incorporation of lentil protein hydrolysates, reflecting the positive influence of higher degrees of hydrolysis. Practically, the results indicate that substituting part of the flour with aquafaba hydrolysates can improve the antioxidant functionality of muffins without fundamentally changing the assay endpoints used to measure radical scavenging; this suggests a route to develop bakery products with enhanced oxidative stability and potential health benefits from dietary antioxidant peptides [83].

## 4. Conclusions

The present study demonstrated that aquafaba hydrolysates, particularly those with higher degrees of hydrolysis, significantly improve the technological, nutritional, and functional qualities of muffin cakes. Enzymatic hydrolysis modified protein profiles, generating low-molecular-weight peptides that enhanced antioxidant capacity, oxidative stability, and textural properties. Cakes enriched with AFHs and ASHs exhibited increased specific volume, protein content, and color quality compared to control samples. Oxitest results confirmed that hydrolysate addition significantly extended induction periods, reflecting improved oxidative resistance. These findings underline aquafaba’s value as a sustainable, vegan by-product that can be transformed into a functional bakery ingredient, supporting eco-friendly and health-conscious product development. However, one limitation of this study is that determining the peptide and amino acid composition released during hydrolysis could more precisely explain the observed effects on the physicochemical and functional characteristics of the cakes. Further research should address this by analyzing peptide profiles, optimizing hydrolysate dosage, exploring alternative hydrolysis strategies, and assessing sensory acceptance across different consumer groups. This work provides a foundation for future studies aiming to integrate aquafaba hydrolysates into a variety of foods, such as innovative bakery products, combining sustainability, functionality, and consumer appeal.

## Figures and Tables

**Table 1:** Some physicochemical and color properties of muffin cake samples.

Sample	Moisture (%)	Protein (%)	Oil (%)	AW (%)	Ash (%)	Specific Volume (mL/g)	Crust Color Properties				Crumb Color Properties			
							L*	a*	b*	ΔE*	L*	a*	b*	ΔE*
**CM**	21.88 ± 0.17 ^a^	9.9 ± 0.19 ^b^	12.83 ± 0.66 ^a^	0.77 ± 0.00 ^a^	1.56 ± 0.11 ^d^	1.04 ± 0.09 ^b^	69.12 ± 0.07 ^a^	4.14 ± 0.21 ^d^	34.25 ± 0.28 ^b^	-	74.23 ± 0.26 ^a^	−0.65 ± 0.02 ^d^	24.32 ± 0.15 ^c^	-
**AIM**	21.57 ± 0.61 ^ab^	12.22 ± 0.02 ^ba^	12.11 ± 0.06 ^a^	0.76 ± 0.00 ^a^	1.70 ± 0.16 ^c^	1.89 ± 0.04 ^a^	63.96 ± 0.33 ^b^	8.08 ± 0.34 ^c^	36.13 ± 0.25 ^a^	3.39 ± 0.06 ^c^	73.24 ± 0.19 ^b^	−0.53 ± 0.01 ^c^	26.92 ± 0.28 ^a^	2.30 ± 0.07 ^a^
**AFHM**	20.99 ± 0.06 ^b^	12.29 ^b^ ± 0.09 ^a^	12.76 ± 0.02 ^a^	0.74 ± 0.01 ^a^	1.90 ± 0.02 ^a^	1.96 ± 0.04 ^a^	58.80 ± 0.65 ^c^	12.50 ± 0.27 ^b^	33.85 ± 0.12 ^b^	4.42 ± 0.02 ^a^	72.66 ± 0.15 ^c^	−0.49 ± 0.02 ^b^	25.97 ± 0.06 ^b^	2.18 ± 0.02 ^a^
**ASHM**	21.06 ± 0.01 ^b^	12.60 ± 0.19 ^a^	12.88 ± 0.15 ^a^	0.74 ± 0.01 ^a^	1.84 ± 0.28 ^b^	2.23 ± 0.17 ^a^	57.06 ± 0.06 ^d^	13.54 ± 0.09 ^a^	29.99 ± 0.45 ^a^	4.11 ± 0.03 ^b^	72.18 ± 0.14 ^c^	−0.43 ± 0.01 ^a^	25.83 ± 0.32 ^b^	1.86 ± 0.01 ^b^

CM: Control muffin cake. AIM: Muffin cake substituted with aquafaba protein isolate. AFHM: Muffin cake substituted with aquafaba hydrolysate obtained by flavourzyme enzyme treatment. ASHM: Muffin cake substituted with aquafaba hydrolysate obtained by savinase enzyme treatment. AW: Water activity. Data are means ± SD of three replicates. Values with different letters in each column are significantly different (*p* < 0.05).

**Table 2:** Power law model parameters of muffin cake batters.

Sample	K (Pa·s^n^)	n	R^2^
**CM**	9.031 ± 0.04 ^a^	0.616 ± 0.01 ^a^	0.994
**AIM**	7.555 ± 0.03 ^b^	0.579 ± 0.00 ^b^	0.989
**AFHM**	6.725 ± 0.06 ^c^	0.625 ± 0.00 ^a^	0.990
**ASHM**	6.390 ± 0.13 ^d^	0.615 ± 0.01 ^a^	0.989

CM: Control muffin cake. AIM: Muffin cake substituted with aquafaba protein isolate. AFHM: Muffin cake substituted with aquafaba hydrolysate obtained by flavourzyme enzyme treatment. ASHM: Muffin cake substituted with aquafaba hydrolysate obtained by savinase enzyme treatment. Data are means ± SD of three replicates. Values with different letters in each column are significantly different (*p* < 0.05).

**Table 3:** Texture Properties of Muffin Cake Samples.

**Sample**	**Hardness (g)**	**Springiness**	**Cohesiveness**	**Gumminess (g)**	**Chewiness (g)**	**Resilience**
**CM**	4043 ± 81 ^a^	0.88 ± 0.01 ^b^	0.58 ± 0.02 ^b^	2323 ± 92 ^a^	2100 ± 72 ^a^	0.26 ± 0.01 ^b^
**AIM**	3511 ± 93 ^b^	0.90 ± 0.01 ^a^	0.66 ± 0.02 ^a^	2313 ± 139 ^a^	2016 ± 26 ^a^	0.31 ± 0.00 ^a^
**AFHM**	2828 ± 58 ^c^	0.90 ± 0.00 ^a^	0.69 ± 0.14 ^a^	1985 ± 101 ^b^	1789 ± 82 ^b^	0.31 ± 0.00 ^a^
**ASHM**	2317 ± 37 ^d^	0.91 ± 0.01 ^a^	0.65 ± 0.01 ^a^	1516 ± 45 ^c^	1380 ± 46 ^c^	0.30 ± 0.00 ^a^
**Sample**	**T_0_**		**T_d_**		**ΔH**	
**CM**	95.18 ± 2.34 ^a^		114.46 ± 3.78 ^a^		329.7 ± 10.87 ^b^	
**AIM**	94.66 ± 2.12 ^a^		114.53 ± 5.30 ^a^		360.9 ± 8.43 ^a^	
**AFHM**	70.53 ± 1.56 ^c^		122.34 ± 6.04 ^b^		270.63 ± 1.59 ^d^	
**ASHM**	75.10 ± 0.96 ^b^		105.49 ± 4.02 ^c^		283.3 ± 4.08 ^c^	

CM: Control muffin cake. AIM: Muffin cake substituted with aquafaba protein isolate. AFHM: Muffin cake substituted with aquafaba hydrolysate obtained by flavourzyme enzyme treatment. ASHM: Muffin cake substituted with aquafaba hydrolysate obtained by savinase enzyme treatment. T_0_: Temperature onset. T_d_: Denaturation temperature. ΔH: Gelatinization enthalpy. Data are means ± SD of three replicates. Values with different letters in each column are significantly different (*p* < 0.05).

**Table 4:** Antioxidant capacity and induction period properties of aquafaba protein hydrolysates and muffin cake samples.

Sample	Antioxidant Capacity *		Induction Period (h:min)
	DPPH	ABTS	
**AI**	11.7 ± 0.44 ^c^	222.58 ± 15.96 ^b^	-
**AFHs**	18.97 ± 0.58 ^b^	257.01 ± 14.90 ^ab^	-
**ASHs**	26.41 ± 1.36 ^a^	294.24 ± 11.92 ^a^	-
**CM**	51.01 ± 1.49 ^D^	262.53 ± 4.7 ^C^	15:08 ± 0.19 ^d^
**AIM**	84.35 ± 2.91 ^C^	481.87 ± 10.9 ^B^	16:32 ± 0.14 ^c^
**AFHM**	105.46 ± 1.33 ^B^	489.74 ± 12.87 ^B^	17:28 ± 0.02 ^b^
**ASHM**	115.46 ± 2.22 ^A^	530.56 ± 5.56 ^A^	18:47 ± 0.12 ^a^

AI: Aquafaba protein isolate, AFHs: Aquafaba hydrolysate obtained by flavourzyme enzyme treatment, ASHs: Aquafaba hydrolysate obtained by savinase enzyme treatment. CM: Control muffin cake, AIM: Muffin cake substituted with aquafaba protein isolate, AFHM: Muffin cake substituted with aquafaba hydrolysate obtained by flavourzyme enzyme treatment, ASHM: Muffin cake substituted with aquafaba hydrolysate obtained by alcalase enzyme treatment. Data are means ± SD of three replicates. Different lowercase letters in the same column indicate statistical significance between the same sample group, and uppercase letters indicate statistical significance between the other sample groups (*p* < 0.05). * Antioxidant capacities values were expressed as mg TE/g sample for aquafaba isolate and hydrolysates and as mg TE/100 g sample for muffin cake samples.

## Data Availability

The original contributions presented in this study are included in the article/Supplementary Materials. Further inquiries can be directed to the corresponding author.

## Supplementary Materials

The following supporting information can be downloaded at: https://www.mdpi.com/article/10.3390/foods14213709/s1, Table S1: Composition and their proportions of ingredients utilized in muffin formulation; Figure S1: Muffin Cake Samples.
