# Supplementary material for: Aquafaba Hydrolysates as Functional Ingredients in Muffin Cakes: Effects on Physicochemical Properties, Quality Attributes, and Antioxidant Activity
Source: Foods. 2025 Oct 30;14(21):3709. doi: 10.3390/foods14213709 (PMC12610219; doi:10.3390/foods14213709)
Supplement: Supplementary file 1 [file foods-14-03709-s001.zip › foods-3937612-supplementary.pdf]

Table S1. Composition and their proportions of ingredients utilized in muffin formulation

Ingredients (%)	CM	AIM	AFHM	ASHM
Egg	90	90	90	90
Sugar	60	60	60	60
Shortening	15	15	15	15
Oil	15	15	15	15
Milk (Whole fat)	60	60	60	60
Wheat flour	100	95	95	95
Baking powder	4.5	4.5	4.5	4.5
Vanilla	1	1	1	1
Aquafaba protein isolate (AI)	0	5	0	0
Aquafaba protein hydrolysate (AFHs)	0	0	5	0
Aquafaba protein hydrolysate(ASHs)	0	0	0	5

CM: Control muffin cake, AIM Muffin cake e substituted with aquafaba protein isolate, AFHM: Muffin cake substituted with aquafaba hydrolysate obtained by flavourzyme enzyme treatment, ASHM: Muffin cake substituted with aquafaba hydrolysate obtained by savinase enzyme treatment, AI: Aquafaba protein isolate, AFH: Aquafaba hydrolysate obtained by flavourzyme enzyme treatment, ASH: Aquafaba hydrolysate obtained by savinase enzyme treatment.

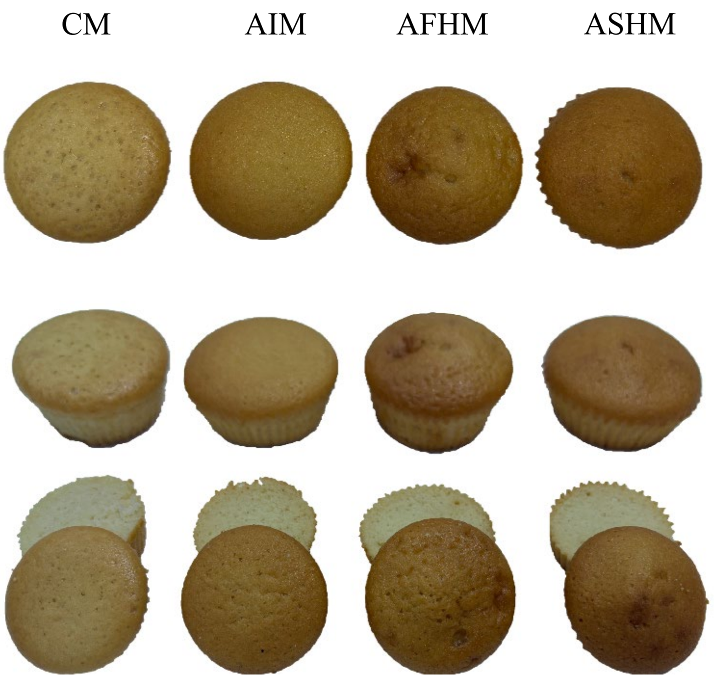

Figure S1. Muffin Cake Samples

CM: Control muffin cake, AIM Muffin cake e substituted with aquafaba protein isolate, AFHM: Muffin cake substituted with aquafaba hydrolysate obtained by flavourzyme enzyme treatment, ASHM: Muffin cake substituted with aquafaba hydrolysate obtained by savinase enzyme treatment,
